# HSP60 plays a regulatory role in IL-1β-induced microglial inflammation via TLR4-p38 MAPK axis

**DOI:** 10.1186/s12974-016-0486-x

**Published:** 2016-02-02

**Authors:** Shalini Swaroop, Nabonita Sengupta, Amol Ratnakar Suryawanshi, Yogita K Adlakha, Anirban Basu

**Affiliations:** National Brain Research Centre, Manesar, Haryana 122051 India; Clinical Proteomics, Institute of Life Sciences, Bhubaneswar, Odhisa 751023 India

**Keywords:** Microglia, IL-1β, HSP60, Inflammation, MAPK, p38, MEK3/6, TLR4

## Abstract

**Background:**

IL-1β, also known as “the master regulator of inflammation”, is a potent pro-inflammatory cytokine secreted by activated microglia in response to pathogenic invasions or neurodegeneration. It initiates a vicious cycle of inflammation and orchestrates various molecular mechanisms involved in neuroinflammation. The role of IL-1β has been extensively studied in neurodegenerative disorders; however, molecular mechanisms underlying inflammation induced by IL-1β are still poorly understood. The objective of our study is the comprehensive identification of molecular circuitry involved in IL-1β-induced inflammation in microglia through protein profiling.

**Methods:**

To achieve our aim, we performed the proteomic analysis of N9 microglial cells with and without IL-1β treatment at different time points. Expression of HSP60 in response to IL-1β administration was checked by quantitative real-time PCR, immunoblotting, and immunofluorescence. Interaction of HSP60 with TLR4 was determined by co-immunoprecipitation. Inhibition of TLR4 was done using TLR4 inhibitor to reveal its effect on IL-1β-induced inflammation. Further, effect of HSP60 knockdown and overexpression were assessed on the inflammation in microglia. Specific MAPK inhibitors were used to reveal the downstream MAPK exclusively involved in HSP60-induced inflammation in microglia.

**Results:**

Total 21 proteins were found to be differentially expressed in response to IL-1β treatment in N9 microglial cells. In silico analysis of these proteins revealed unfolded protein response as one of the most significant molecular functions, and HSP60 turned out to be a key hub molecule. IL-1β induced the expression as well as secretion of HSP60 in extracellular milieu during inflammation of N9 cells. Secreted HSP60 binds to TLR4 and inhibition of TLR4 suppressed IL-1β-induced inflammation to a significant extent. Our knockdown and overexpression studies demonstrated that HSP60 increases the phosphorylation of ERK, JNK, and p38 MAPKs in N9 cells during inflammation. Specific inhibition of p38 by inhibitors suppressed HSP60-induced inflammation, thus pointed towards the major role of p38 MAPK rather than ERK1/2 and JNK in HSP60-induced inflammation. Furthermore, silencing of upstream modulator of p38, i.e., MEK3/6 also reduced HSP60-induced inflammation.

**Conclusions:**

IL-1β induces expression of HSP60 in N9 microglial cells that further augments inflammation via TLR4-p38 MAPK axis.

**Electronic supplementary material:**

The online version of this article (doi:10.1186/s12974-016-0486-x) contains supplementary material, which is available to authorized users.

## Background

Neuroinflammation being the first line of defense of the central nervous system (CNS) provides innate immunity to the brain and spinal cord. It can be evoked by various factors ranging from bacterial infections to neurodegenerative disorders that mediate acute and chronic inflammations, respectively [1–3]. In addition, it may also be caused by an autoimmune response such as multiple sclerosis or in response to toxins and nerve agents [4, 5]. Inflammation in the CNS, however, acts as a double-edged sword, as on one hand, it serves to protect the CNS from infection and neuronal injury but on the other hand, an exaggerated inflammatory process may lead to further neurodegeneration and neuronal loss [6].

Among several cell types implicated in inflammation, microglia play a major role in innate immune response [7, 8]. They get activated in response to hazardous stimuli, such as brain injury, immunological stimuli such as endotoxins, and other insults to the brain [4, 9, 10]. Upon activation, these cells release various pro- and anti-inflammatory cyto-chemokines (for example, macrophage chemoattractant protein-1 (MCP-1), IL-1β, IL-6, and TNF-α) [11, 12], pro-inflammatory enzymes (inducible nitric oxide synthase (iNOS) and cyclooxygenase-2 (COX2)) [13], and reactive oxygen species [14] to combat infection. However, an exaggerated microglia response can be detrimental to the normal functioning of the CNS [4]. Abnormal microglial activation is also attributed to the pathology of the several neurodegenerative diseases including Alzheimer’s disease (AD) [12], Parkinson’s disease (PD) [15], multiple sclerosis [4], psychiatric disorders such as stress, depression, and schizophrenia [16], and metabolic syndromes such as obesity and type 2 diabetes [17].

Among the various factors secreted by activated microglia, IL-1β is a prominent pro-inflammatory cytokine which plays a crucial role in the progression of chronic neurodegenerative diseases as well as acute neuroinflammatory conditions [18–20]. Once secreted by the activated microglia and astrocytes [21], it can further stimulate its own production in an autocrine and/or, paracrine fashion by binding to its cognate IL-1 receptors (IL-1Rs) [21, 22], this leads to a constitutive expression of IL-1β which further amplifies the inflammatory signal. After binding, it can upregulate the production of other pro-inflammatory cytokines, prostaglandins, and other toxic mediators like ROS, by starting a vicious cycle of biochemical pathways, and is therefore, considered as the “master regulator of inflammation” [23, 24]. However, the molecular signaling underlying IL-1β-induced inflammation during microglial activation is not fully understood.

Heat shock proteins (HSPs), represent a collection of highly conserved proteins constitutively expressed in most cells under cellular stress conditions like, nutrient deprivation or mechanical damage and are considered as endogenous danger signals to the immune system [25, 26]. One of the important mitochondrial molecular chaperones is HSP60 which contributes to the proper folding of the proteins and restoration of the tertiary structure of the misfolded or denatured proteins [27]. Interestingly, HSP60 has been reported to play immunomodulatory role in case of various infections [28–30]. In addition, several studies suggest that HSP60 serves as an endogenous signal of injury in the CNS by activating microglia after its release from injured neurons and by binding to toll-like receptor 4 (TLR4) in a myeloid differentiation factor 88 (Myd88) dependent pathway [31, 32]. Intrathecal HSP60 mediates neurodegeneration and demyelination through a TLR4-Myd88 dependent pathway [33]. Despite its chaperone activities, HSP60 can also appear in extracellular milieu where it elicits a potent pro-inflammatory response in the peripheral immune system [34]. Besides its chaperone and immunomodulatory roles, the function of HSP60 in response to Il-1β-induced inflammation in microglial cells is unknown.

As understanding the mechanism of IL-1β-induced inflammation in microglia is of considerable importance in neuroinflammation biology, hence we set out to investigate molecular circuitry underlying IL-1β-induced inflammation in microglia and how HSP60 modulates this circuitry. Herein, we demonstrate that HSP60 aggravates IL-1β-induced inflammation in microglia via TLR4 receptors and MAPK signaling pathway. Our results further suggest that p38 MAPK is the major player in HSP60-induced inflammation which acts following the activation of MEK3/6.

## Methods

### Animal experiments

P10 (postnatal day 10) BALB/c mice of either sex were intraperitoneally (i.p.) injected with 50 μl of 10 ng/g body weight of IL-1β dissolved in 1× phosphate-buffered saline (PBS) every 24 h for different durations (1, 3, and 5 days) as described elsewhere [35], while control-treatment group received the same volume of the carrier (1× PBS). Groups of three mice were sacrificed at each time point either for protein or mRNA isolation. P0–P2 (postnatal days 0–2) BALB/c mice of either sex were procured for primary microglial culture. Animals were handled in strict accordance with good animal practice as defined by the Committee for the Purpose of Control and Supervision of Experiments on Animals (CPCSEA) and the Ministry of Environment and Forestry, Government of India. The Institutional Animal Ethics Committee (IAEC) of the National Brain Research Centre approved the study protocol (NBRC/IAEC/2013/77 and NBRC/IAEC/2012/70).

### Cell culture

Primary microglial cells were isolated from BALB/c mouse pups (postnatal days 0–2) as reported previously [36]. Briefly, the whole brain cortex was dissected from the mouse brain, and the meninges were peeled off under a dissecting microscope. Tissue was digested using trypsin-DNase I solution at 37 °C, with a brief mechanical dissociation to obtain a cell suspension. The cell suspension was passed through 130-μm cell strainers, and the supernatant was centrifuged at 800 rpm for 10 min to obtain a cell pellet. Cells were seeded in 75-cm^2^ tissue culture flasks at a density of 2 × 10^5^ viable cells/cm^2^ in complete MEM (supplemented with 10 % fetal bovine serum, 100 units/ml penicillin, 100 μg/ml streptomycin, 0.6 % glucose, and 2 mM glutamine). The exhausted media was changed every 2 days with fresh complete MEM, until the mixed glial culture became confluent. On day 12, the flasks were shaken on an Excella E25 orbital shaker (New Brunswick Scientific, NJ, USA) at 250 rpm for 90 min at 37 °C to dislodge microglial cells. The non-adherent cells thus obtained were plated in bacteriological petridishes for 90 min to allow microglial cells to adhere. The adherent cells were then scraped, centrifuged, and plated in chamber slides at 8 × 10^4^ viable cells/cm^2^ and incubated at 37 °C for further experiments.

Mouse microglial cell line N9 was a kind gift from Prof. Maria Pedroso de Lima, Center for Neuroscience and Cell Biology, University of Coimbra, Portugal. The cell lines were grown at 37 °C in RPMI-1640 supplemented with 10 % fetal bovine serum, 100 units/ml penicillin, and 100 μg/ml streptomycin. IL-1β treatment was given to N9 cells at a dose of 5 ng/ml at different time points (3, 6, and 12 h) in vitro. All the reagents related to cell culture were obtained from Sigma-Aldrich, St. Louis, USA, unless otherwise stated.

### IL-1β, MAP kinase inhibitors and TLR4 inhibitor treatment

Recombinant mouse IL-1β was purchased from R&D systems and used to induce inflammation. Cells were seeded in 60 mm^2^ plates and specific MAP kinase inhibitors including U0126 (ERK inhibitor, Calbiochem), SP600125 (JNK inhibitor, Sigma-Aldrich), SB203580 (p38 inhibitor, Sigma-Aldrich) were used at 10 μM concentration 1 h prior to IL-1β treatment. CLI-095 (TLR4 inhibitor, Invivogen) was used at 5 and 10 μM concentration 2 h prior to IL-1β treatment.

### Knockdown and overexpression studies

Knockdown studies were performed using endonuclease-prepared short interfering RNA (esiRNA) against mouse HSP60 (EMU151751) and scrambled esiRNA (enhanced green fluorescent protein (eGFP)) (sense, 5′-GTG AGC AAG GGC GAGGAG CTG TTC ACC GGG GTG GTG CCC ATC CTG GTC GAG CTG GA-3′) and were purchased from Sigma-Aldrich. A total of 6 pM HSP60 or 8 pM MEK3/6 esiRNA were used for transfection using Lipofectamine 2000 (Invitrogen, Carlsbad, CA, USA) according to the manufacturer’s protocol. After 24 h of transfection, cells were further treated with IL-1β for 3 h and processed for immunoblotting and cytokine bead array. Overexpression of HSP60 in N9 cells was achieved by transfection of mouse HSP60 plasmid clone (MC206740, OriGene) in 60 mm^2^ plates using lipofectamine 2000 (Invitrogen, Carlsbad, CA, USA) according to the manufacturer’s protocol. The media was changed after 6 h of transfection, and cells were further kept for 24 h to allow overexpression of the cloned HSP60 gene. The control cells were transfected with pCMV6 empty plasmid vector.

### Proteomic profiling

#### Sample preparation and two-dimensional gel electrophoresis (2-DE)

2-DE was performed as described earlier [37]. Untreated control and treated N9 cells were lysed in buffer containing 8 M urea, 2 % (*w*/*v*) CHAPS, 0.2 % sodium orthovanadate, and protease inhibitor cocktail (Sigma-Aldrich, USA). Samples were sonicated and centrifuged at 20,000*g* for 30 min at 4 °C to remove debris. The proteins were further precipitated using trichloroacetic acid (TCA) at 4 °C overnight followed by centrifugation at 20,000*g* at 4 °C.

The protein pellet was resuspended in sample rehydration buffer (8 M urea, 2 % *w*/*v* CHAPS, 15 mM DTT, and 0.5 % *v*/*v* IPG buffer pH 3–10). For the first dimension, 500 μg of each protein sample was solubilized in 150 μl of rehydration solution and IPG strips (7 cm, pH 4–7, linear) were rehydrated for 16 h with rehydration buffer containing sample. Isoelectric focusing was carried out at 10000 V-hr at 20 °C on a Protean i12™ IEF Cell (Bio-Rad, USA). After focusing, the strips were incubated for 10 min in 5 ml of equilibration buffer I (6 M urea, 30 % *w*/*v* glycerol, 2 % *w*/*v* SDS, and 1 % *w*/*v* DTT in 50 mM Tris/HCl buffer, pH 8.8) followed by equilibration buffer II (6 M urea, 30 % *w*/*v* glycerol, 2 % *w*/*v* SDS, and 4 % *w*/*v* iodoacetamide in 375 mM Tris/HCl buffer, pH 8.8). The second-dimensional separation was conducted on 1.5-mm thick 10 % polyacrylamide gels using the Protean-II electrophoresis cell (BioRad, Hercules, CA, USA).

#### Protein visualization and image analysis

Protein spots were visualized by staining with Coomassie Brilliant Blue G-250, and the gel images were captured by LI-COR odyssey infra-red imager (LI-COR Biosciences, USA). Four biological replicates each with two analytical replicate (*n* = 8) images per dataset (untreated control versus different time points of IL-1β-treated N9 cells) were used for automatic spot detection using PD Quest 2D Analysis Software (Hercules, CA, USA). Spot intensities were normalized by total valid spot intensities and mean of values from duplicate analytical gels from four biological replicates were subjected to paired *t* test analysis using GraphPad Prism software. Protein spots showing altered expression between control and experimental groups (|ratio| ≥ 1.5, *p* ≤ 0.05) were marked and excised by use of thin-walled PCR tubes (200 μl) and appropriately cut at the bottom with a fresh surgical scalpel blade. Care was taken not to contaminate the spots with adjoining proteins or with skin keratin.

#### Mass spectrometry analysis and database searching

Proteins were identified by mass spectrometry (MS) using an AB Sciex MALDI TOF/TOF 5800 (AB Sciex, CA, USA) at Institute of Life Sciences, Bhubaneswar, after washing and in-gel trypsin digestion of gel spots. All MS and MS/MS spectra were simultaneously submitted to ProteinPilot software version 3.0 (Applied Biosystems) for database searching using Mascot search engine against UniprotKB-Swissprot database containing 544996 sequences with the taxonomy group of *Mus musculus*. Search parameters were as follows: trypsin digestion with one missed cleavage, variable modifications (oxidation of methionine and carbamidomethylation of cysteine), and the peptide mass tolerance of 100 ppm for precursor ion and mass tolerance of ±0.8 Da for fragment ion with +1 charge state. Results obtained from database search were further analyzed. Proteins from *M. musculus* species with significant Mowse scores and more than one unique peptide were identified and used for further study as shown in Table S1 in the Additional file 1).

### Functional analysis using GeneCodis and String Software

The list of differentially expressed genes/proteins obtained after the proteomic analysis of IL-1β-treated N9 cells were also imported into the GeneCodis software. In our analysis, we used the default settings of GeneCodis, which employs hypergeometric test for calculating *P* values and false-discovery rate for *P* values correction [38].

We studied interactomes of differentially expressed genes/proteins using Search Tool for the Retrieval of Interacting Genes/Proteins (STRING) database. For this, we first generated first order protein-protein interaction network of the identified proteins with the help of STRING database [39], at low confidence value (0.150), to identify highest possible connections and applied highest degree of Markov Cluster Algorithm (MCL) clustering to determine different clusters.

### Western blotting

Untreated and treated N9 cells were lysed in buffer containing 1 % Triton-X-100, 10 mM Tris (hydroxymethyl) aminomethane-Cl (pH 8.0), 150 mM sodium chloride, 0.5 % octylphenoxypolyethoxyethanol (Nonidet P-40), 1 mM ethylenediaminetetraacetic acid, 0.2 % ethylene glycol tetraacetic acid, 0.2 % sodium orthovanadate, and protease inhibitor cocktail (Sigma-Aldrich). The lysate was centrifuged at 12,000*g* for 30 min at 4 °C and supernatant was collected.

For western blotting of the proteins secreted in the media, the proteins present in the used culture media were precipitated overnight by using 1/4th volume of TCA at 4 °C and centrifuged at 20,000*g*. The pellet was washed with acetone and air dried and resuspended in 2 % urea-CHAPS before loading in 10 % SDS polyacrylamide gel. Western blotting was performed as previously described [24]. Following primary antibodies were used: Anti HSP60, Anti-MEK3/6 (Abcam), phospho- and total-ERK1/2, phospho- and total JNK1/2, phospho- and total p38 (Cell Signaling), Anti-TLR4 and phospho-MEK3/6 (Santa Cruz Biotechnology), and β-actin (Sigma-Aldrich). Secondary antibodies were horseradish peroxidase labeled. The blots were developed using chemiluminescence reagent (Millipore) in ChemiGenius Bioimaging System (Syngene, Cambridge, UK). The images were captured and analyzed using the GeneSnap and GeneTools software, respectively, from Syngene. The protein levels were normalized to β-actin levels. The fold change with respect to control cells was then calculated based on integrated density values (IDV). All experiments were repeated at least three times and representative blots are shown.

### Quantitative real-time PCR (qRT-PCR)

Total RNA from N9 cells and mouse brains was isolated using TRI Reagent (Sigma-Aldrich), and reverse transcription was carried out using an Advantage RT-for-PCR kit (Clontech Laboratories). Real-time PCR was done using power SYBR Green PCR master mix (Applied Biosystems, Foster City, CA, USA) in i7 real-time PCR instrument (Applied Biosystems) as described previously [36]. Sequence for primers used for real-time PCR is given in Additional file 1: Table S2. GAPDH mRNA was used as endogenous control for normalization. Relative quantitation of gene expression was carried out using the Pfaffl method [40].

### Cytokine bead array (CBA)

Fifty microgram protein from the cell and brain lysate was used for the quantification of the levels of cytokines in control and treated condition. CBA was performed using a mouse CBA kit (BD Biosciences, Franklin Lakes, NJ, USA) according to the manufacturer’s instructions. The beads coated with interleukin 6 (IL-6), tumor necrosis factor alpha (TNF-α), and monocyte chemotactic protein 1 (MCP-1) were mixed with 50 μg cell lysates and standards, to which fluorescent dye phycoerythrine (PE) was added. The experiment was performed in triplicates as described [41], and data was analyzed using BD CBA software (Becton, Dickinson, San Diego, CA, USA). The concentrations of various cytokines were expressed as fold change with respect to control.

### Immunofluorescence analysis

Immunofluorescence was performed as described previously [24]. Primary mouse microglial cells as well as N9 murine microglial cells were stained with anti-HSP60 (Abcam) and Iba-1 (Millipore) antibodies. Fluorescein isothiocyanate (FITC)-conjugated secondary antibody was used with mounting medium containing 4,6-diamidino-2-phenylindole (Vector Laboratories, Burlingame, CA, USA). The fluorescence images were captured using Zeiss apotome microscope (Carl Zeiss MicroImaging GmbH, Göttingen Germany; Scale bar—20 μm) at ×40 magnification under corresponding excitation and emission wavelengths.

### Co-immunoprecipitation assay

Treated and untreated N9 murine microglial cells were lysed with cell lysis buffer (50 mM Tris buffer, pH 7.4, containing 150 mM NaCl, 5 mM EDTA, 1 % NP-40) with freshly added protease inhibitors (1 mg/ml aprotinin, 1 mg/ml leupeptin, 1 mg/ml pepstatin, and 1 mM PMSF) and phosphatase inhibitors (20 mM NaF and 1 mM orthovanadate). Lysates were co-immunoprecipitated with 5 μg of anti-HSP60 antibody (Abcam) for overnight at 4 °C and incubated with protein A Sepharose beads (Sigma) for 2 h at 4 °C. The immunocomplexes were then washed and probed by western blotting using anti-TLR4 antibody as well as anti-HSP60 antibody.

### Statistical analysis

Data are represented as the mean ± standard deviation (SD) from at least three independent experiments. The data was analyzed statistically by paired two-tailed Student’s *t* test. *p* < 0.05 were considered significant.

## Results

### IL-1β administration induces inflammation in microglia both in vitro and in vivo

**Fig. 1:**
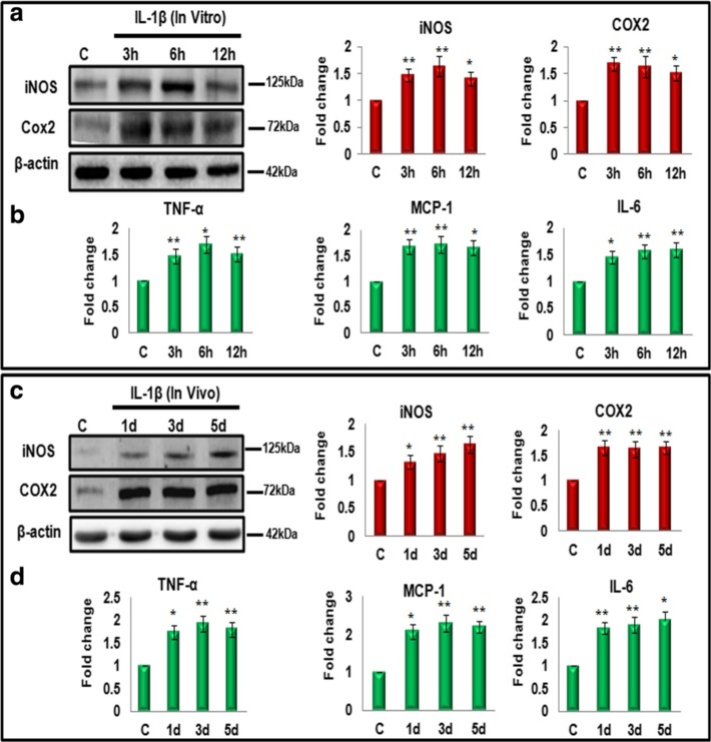
IL-1β induces inflammation both in vitro and in vivo. **a** *Left panel* shows the representative western blot images of iNOS and COX2 from N9 cell lysates at 3, 6, and 12 h after 5 ng/ml of IL-1β treatment. *Right panel* shows the bar diagrams which represent mean fold change in the levels of iNOS and COX2 after IL-1β treatment with respect to control. **b** *Bar diagrams* represent the mean fold change after CBA analysis of pro-inflammatory cytokines, i.e., TNF-α, MCP-1, and IL-6 after IL-1β treatment at 3, 6, and 12 h. **c** *Left panel* shows the representative western blot images of iNOS and COX2 from P10 BALB/c mice brain after IL-1β treatment (10 ng/g of body weight, intraperitoneally injected) for different time periods (1, 3, and 5 days). *Right panel* shows the bar diagrams which represent mean fold change in the level of respective proteins in comparison to control at different time points. One hundred microgram of the protein was loaded for western blot (**a** and **c**) and the levels of iNOS and COX2 were normalized with β-actin. **d** CBA analysis of pro-inflammatory cytokines (TNF-α, MCP-1, and IL-6) at different time points of IL-1β treatment. Data represent mean ± SD from three different sets of experiments. *p < 0.05, **p < 0.01 in comparison to untreated control condition

IL-1β, being the master regulator of inflammation, is well known to induce inflammation in microglia by triggering a cascade of molecular pathways leading to the activation of microglia by the production of pro-inflammatory molecules and cyto-chemokines [24]. We first assessed the extent of IL-1β-induced inflammation in microglia in vitro by treating N9 murine microglial cells with IL-1β and determined the levels of pro-inflammatory enzymes (iNOS and COX2) and pro-inflammatory cytokines (TNF-α, MCP-1, and IL-6). The N9 murine microglial cells were treated with 5 ng/ml IL-1β for 3, 6, and 12 h. Consistent with previous reports, a significant increase in the expression of pro-inflammatory markers, iNOS, and COX2 was observed at 3, 6, and 12 h of treatment in microglial cells as compared to control cells (Fig. 1a). Further, we observed significant increase in the levels of pro-inflammatory cytokines (TNF-α, MCP-1, and IL-6) in IL-1β-treated cells as revealed by cytokine bead array (CBA) (Fig. 1b), thus confirming the role of IL-1β in inducing inflammation in microglial cells.

In addition, we checked for the inflammatory effect of IL-1β in vivo also (Fig. 1c, d). For this, P10 (postnatal day 10) BALB/c mice were injected with 10 ng/g body weight of IL-1β for 1, 3, and 5 days as described elsewhere [35]. Control group received the same volume of the carrier (1× PBS). Further, we checked the expression of pro-inflammatory enzymes and cytokines to assess inflammation. We observed time dependent increase in iNOS and consistent increase in COX2 protein levels (Fig. 1c), as well as, in TNF-α, MCP-1, and IL-6 levels (Fig. 1d) at 1, 3, and 5 days of IL-1β treatment in mouse brain. This further confirmed the inflammatory role of IL-1β in mouse brain, thus strengthening our in vitro data.

### Identification of global host proteome response post IL-1β administration in N9 microglial cells

**Fig. 2:**
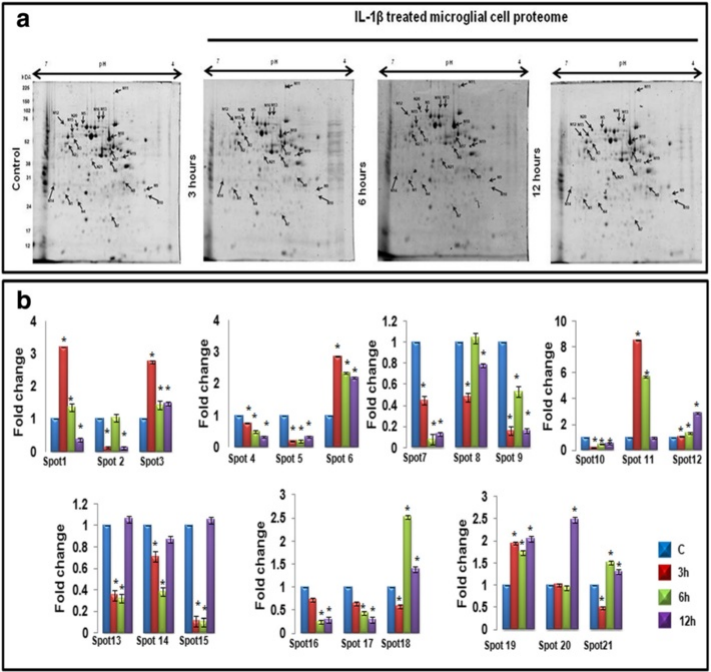
Proteomic analysis of control and IL-1β-treated N9 microglial cell lysates at different time points. **a** Equal amount of total protein from control and IL-1β-treated N9 cells (3, 6, and 12 h) were separated on an immobilized linear pH gradient IPG strips (4.0–7.0) and then by second dimension on 10 % SDS-PAGE. Spots showing differential expression were marked and excised and identified by MALDI TOF/MS and database searches. The spots were labeled on the gel according to the numbers presented in Additional file 1: Table S1. **b** *Bar diagrams* represent relative fold changes in differentially expressed proteins in IL-1β-treated N9 microglial cells with respect to control. Total 21 spots were taken. Spot intensities were normalized by total valid spot intensities and mean of values from duplicate analytical gels from four biological replicates and were subjected to paired *t* test analysis. Protein spots showing altered expression between control and experimental groups (|ratio| > = 1.5, *p* ≤ 0.05) were marked and excised. *p < 0.05. Data represented are means ± SD of four independent experiments

The microglial proteome has not been analyzed in response to the leading cytokine IL-1β till now; therefore, we set out to identify differentially expressed proteins in response to IL-1β in microglial cells. N9 murine microglial cells were treated with IL-1β (5 ng/ml) to induce inflammation and proteomic analyses of control, and treated N9 cells was done at different time points (3, 6, and 12 h) followed by 2D-gel electrophoresis (Fig. 2a). 2D-gel images for control versus IL-1β-treated N9 cells of different time points were quantitatively analyzed using PD Quest software as shown in Fig. 2b. In total, 21 spots were found to be differentially regulated. These 21 protein spots showing differential expression of 1.5-fold or greater (*p* < 0.05) were excised, trypsin digested, and identified by MALDI TOF/TOF MS and MS/MS analysis, which revealed seventeen different types of proteins. Among them, nine proteins were significantly upregulated while the rest were found to be downregulated. The observed MW and pI values of the protein spots on 2-DE gels were compared with the theoretical MW and pI values of the corresponding proteins (Additional file 1: Table S1), and most experimental values were found to be close to theoretical values, indicating unambiguous identification except a few. The list of all identified proteins along with their *P* values and the average ratio is given in Additional file 1: Table S1.

To investigate possible biological functions of differentially regulated proteins, we performed in silico analysis using GeneCodis3 software [42] which revealed eleven significant molecular functions. Out of these functions, unfolded protein binding was one of the highest rated molecular functions (Additional file 1: Figure S1). We further did interactome studies with the help of STRING database to find out the proteins playing key role in the interactome developed from the identified proteins [39]. Out of these, HSP60 (HSPD1) was found to be present in the biggest cluster of proteins and turned out to have highest numbers of interactions with other proteins of the interactome (Additional file 1: Figure S2).

### IL-1β administration increases HSP60 expression both in vitro and in vivo

**Fig. 3:**
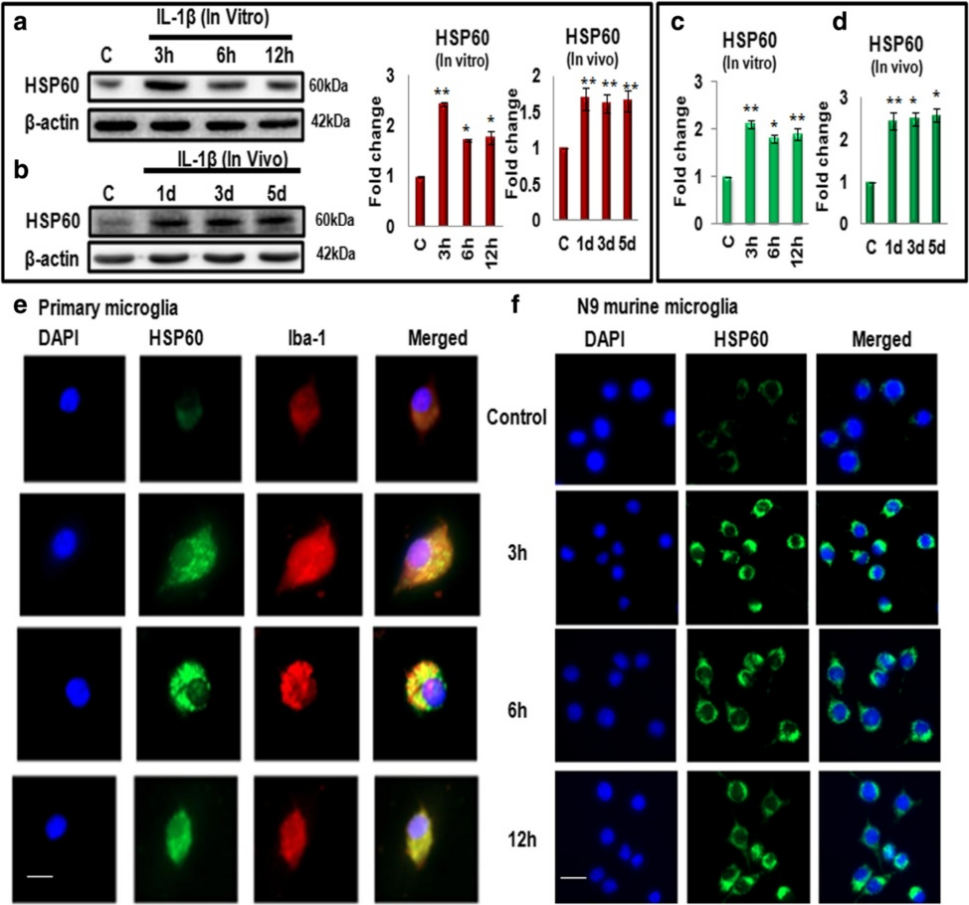
IL-1β induces the expression of HSP60 in vitro, in vivo, and in primary microglial culture. **a**, **b** *Left panel* depicts the representative western blot image showing increase in the level of HSP60 at different time points after IL-1β treatment in N9 cells (in vitro) (**a**) and in BALB/c mice brains (in vivo) (**b**), respectively. *Right panel* represents the bar diagrams which depict mean fold change in the levels of HSP60 with respect to control treated group. Thirty microgram protein was loaded for western blot, and β-actin served as a loading control. **c**, **d** Quantitative real-time PCR analysis of the transcript level of HSP60 after treatment with IL-1β at different time points in N9 murine microglial cells (**c**) and in BALB/c mice brain (**d**). GAPDH was used for the normalization. **e**, **f** Immunostaining of HSP60 in primary microglia (**e**) and N9 murine microglial cells (**f**) using specific antibodies as described in methods. Nuclei were counterstained with the DNA-binding dye DAPI. Images were captured using Zeiss apotome fluorescence microscope (Scale bar—20 μm; magnification—×40). Representative of three independent experiments is shown here (**a**, **b**, **e**, **f**) (*n* = 3). *p < 0.05, **p < 0.01 in comparison to control values. Data represented are mean ± SD of three independent experiments

HSP60 is a molecular chaperone of mitochondria, which plays an important role in neuron-glia crosstalk during neurodegeneration [31], and it has also been detected in our interactome studies as one of highly interacting proteins; therefore, we next focused on HSP60 and set out to investigate the role of HSP60 in IL-1β-induced inflammation. The expression of HSP60 was determined both by western blotting (Fig. 3a, b) and quantitative real-time PCR (Fig. 3c, d) at different time points of IL-1β treatment both in vitro (in N9 cells) and in vivo (in mice brain). As shown in Fig. 3a–d, the protein as well as transcript levels of HSP60 were increased significantly as compared to control in response to IL-1β treatment at different time points both in vitro (Fig. 3a, c) and in vivo (Fig. 3b, d).

Further, using double immunostaining, we observed that within 3 h of IL-1β treatment, the primary microglial cells exhibited a transformation from “resting” state, with basal levels of Iba1 expression (control, upper panel, Fig. 3e) to an “activated” state with increased Iba1 expression (3, 6, and 12-h treatment groups, lower panels, Fig. 3e). In addition, expression of HSP60 increased significantly after IL-1β treatment in the primary microglial cells (Fig. 3e) as well as N9 cells (Fig. 3f) as compared to control cells as witnessed by co-localization of HSP60 (green) with Iba1 (red) (Fig. 3e, f). These results justify and strengthen our proteomics analysis.

### Microglial activation through IL-1β administration leads to the secretion of HSP60 in extracellular milieu

**Fig. 4:**
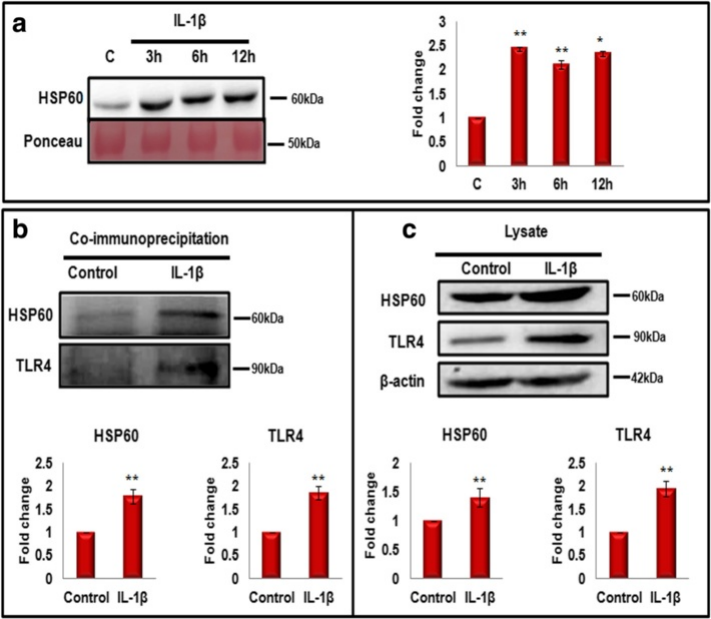
HSP60 is secreted by microglia in the surrounding medium and interacts with TLR4 during inflammation. **a** N9 murine microglial cells were treated with IL-1β for different time periods (3, 6, and 12 h), and the proteins in the used medium were precipitated with trichloroacetic acid (TCA). Western blotting was performed to determine the levels of HSP60 in secretome. Normalization was performed with Ponceau-stained bands. *Right panel* shows the bar diagram representing mean fold changes in the level of HSP60 with respect to control N9 cells. Twenty microgram of the secreted protein was loaded for western blot of HSP60. **b** Co-immunoprecipitation analysis of the interaction between HSP60 and TLR4 in cells treated with IL-1β for 3 h. Whole-cell extracts (500 μg) of untreated and treated N9 microglial cells were immunoprecipitated with anti-HSP60 and anti-IgG antibodies and analyzed by western blot analysis with anti-TLR4 antibody (*left panel*). *Right panel* (**c**) shows the western blots with 100 μg of lysates for the detection of total HSP60, TLR4, and β-actin in the IL-1β-treated cells as compared to control cells. *Lower panel* (**b**, **c**) represents bar diagrams which depict mean fold change in the expression of HSP60 and TLR4 in comparison to control in immunoprecipitate (**b**) and lysate (**c**), respectively. Representative blots of the three independent experiments are shown here. *p < 0.05, **p < 0.01 in comparison to control values. Data represented are mean ± SD of three independent experiments

Literature suggests that HSP60 can be released by the damaged or injured CNS cells and can further activate microglia [31, 43]. Therefore, we hypothesized that HSP60 could also be secreted by the activated microglia to further aggravate the immune response in CNS. To test the hypothesis, we next assessed HSP60 levels in secretome of microglial cells after IL-1β treatment. The proteins present in the media of control and IL-1β-treated N9 cells were precipitated by adding 1/4th volume of trichloroacetic acid (TCA) and were separated by western blotting. Surprisingly, HSP60 levels were increased significantly in the secreted media of IL-1β-treated cells at all time points (3, 6, and 12 h) with respect to control (Fig. 4a), suggesting that IL-1β not only increases the expression of intracellular HSP60 in microglia, but also induces the secretion of HSP60 by microglia in the surroundings.

### Interaction among HSP60 and toll-like receptor 4 (TLR4) and the role of TLR4 in IL-1β-induced inflammation

Reports further suggest that secreted HSP60 serves as a signal of CNS injury by activating microglia through TLR4-MyD88 dependent pathway [31]. To check whether HSP60 secreted by microglia in response to IL-1β treatment binds with TLR4, we determined the interaction between HSP60 and TLR4 using co-immunoprecipitation technique. Five hundred microgram of N9 microglial cellular extract was precipitated with HSP60 antibody, and the blots were probed for TLR4 as well as for HSP60. We found the expression of TLR4 in the immunoprecipitate that was pulled using HSP60 antibody (Fig. 4b). Further, increase in the levels of HSP60 was accompanied with the increase in TLR4 in treated N9 murine microglial cells indicated a possible interaction between HSP60 and TLR4 (Fig. 4c).

**Fig. 5:**
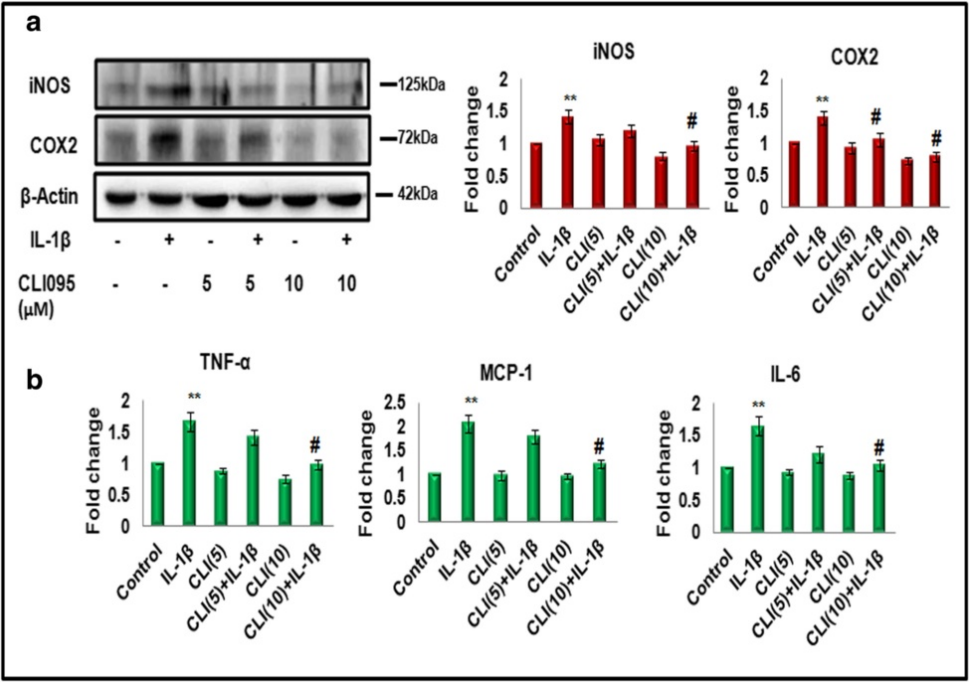
Inhibition of TLR4 results in reduced inflammation in microglia in response to IL-1β treatment. Cells were cultured in the presence or absence of TLR4 inhibitor (CLI-095) at different concentrations (5 and 10 μM), 2 h prior to IL-1β treatment. **a** *Left panel* depicts western blot analysis of iNOS and COX2 showing effect of TLR4 inhibitor on IL-1β-induced inflammation in microglia, *right panel* represents the bar diagrams which reflect mean fold change in expression as compared to control. One hundred microgram protein was loaded for western blots of iNOS and COX2, and β-actin was used as a loading control. The blots are representative of three independent experiments. **b** Effect of inhibition of TLR4 on pro-inflammatory cytokines (TNF-α, MCP-1, and IL-6) was assessed by CBA. *Bar diagrams* are representative of three independent experiments with similar results. Data represented are mean ± SD of three independent experiments. *p < 0.05, **p < 0.01 in comparison to control values and ^#^ *p* < 0.01 in comparison to IL-1β treatment

To investigate the role of TLR4 in IL-1β-induced inflammation, we inhibited TLR4 signaling by using specific TLR4 signaling inhibitor (CLI-095, InvivoGen) in N9 murine microglial cells as described in methods. The levels of iNOS and COX2 were checked by western blot and the pro-inflammatory cytokines (MCP-1, TNF-α, and IL-6) were assessed by CBA. As shown in Fig. 5a, b, the levels of iNOS, COX2, and pro-inflammatory cytokines decreased significantly in presence of 10 μM dose of TLR4 inhibitor in N9 murine microglial cells (Fig. 5a, b). TLR4 inhibitor also reduces the levels of inflammatory molecules induced by IL-1β (Fig. 5a, b). These results suggest that TLR4, in addition to IL-1R1 (specific receptor of IL-1β), plays an important role in IL-1β-mediated signaling in microglia.

### Effect of knockdown and overexpression of HSP60 on inflammation

**Fig. 6:**
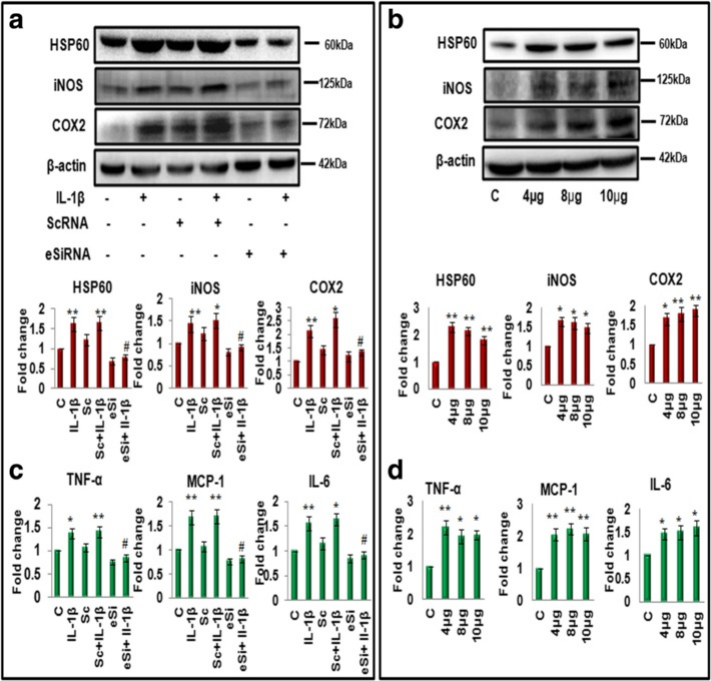
HSP60 plays a modulatory role in induction of inflammation in microglia. Knockdown as well as overexpression of HSP60 was done in N9 murine microglial cells by transfection of specific eSiRNA against HSP60 mRNA (Fig. 6a, c) and mouse HSP60 cDNA clone (Fig. 6b, d), respectively, to check subsequent effects on pro-inflammatory factors. **a** *Left upper panel* shows representative western blot image of HSP60, iNOS and COX2 in the presence of HSP60 esiRNA (6pM) or scrambled esiRNA and/or IL-1β in N9 microglial cells. *Left lower panel* shows the bar diagram which represent mean fold change in the levels of HSP60, iNOS, and COX2 with respect to control. One hundred microgram protein was loaded for western blots of iNOS and COX2, and β-actin was used as a loading control. **b** *Right upper panel* shows the effect of overexpression of HSP60 on iNOS and COX2 by western blotting. *Lower panel* shows the bar diagram which represent fold change in the levels of HSP60, iNOS and COX2 with respect to control. One hundred microgram protein was loaded for western blots of iNOS and COX2 and 20 μg for HSP60. β-actin was used as a loading control. The blots are representative of three independent experiments. **c**, **d** CBA analysis of pro-inflammatory cytokines TNF-α, MCP-1, and IL-6 in presence of HSP60 esiRNA (**c**) and mouse HSP60 cDNA clone (**d**). Data represented are mean ± SD of three independent experiments. *p < 0.05; **p < 0.01 in comparison to control values and ^#^ *p* < 0.01 in comparison to IL-1β treatment

To assess the effect of HSP60 on inflammation, various inflammatory molecules were studied after the knockdown as well as overexpression of HSP60 in N9 microglial cells in vitro. For knockdown studies, N9 microglial cells were transfected with 6pM HSP60 eSiRNA and scrambled eGFP eSiRNA and the knockdown of HSP60 was confirmed by western blotting (Fig. 6a). As shown in Fig. 6, the levels of iNOS, COX2 (Fig. 6a), and pro-inflammatory cytokines (MCP-1, TNF-α, and IL-6) (Fig. 6c) decreased significantly in N9 microglial cells in presence of HSP60 eSiRNA, as compared to scrambled eGFP eSiRNA-transfected cells and this reduction was persistent even after the addition of IL-1β.

In contrast, we did overexpression of HSP60 in N9 cells using mouse HSP60 cDNA clone at different concentrations (4, 8, and 10 μg), and the over expression was confirmed by western blot (Fig. 6b). The levels of inflammatory molecules including iNOS, COX2 (Fig. 6b), and pro-inflammatory cytokines (MCP-1, TNF-α, and IL-6) (Fig. 6d) increased significantly after overexpression of HSP60 alone without IL-1β treatment. These results suggest that HSP60 plays a modulatory role in IL-1β-induced inflammation in microglia.

### TLR4 plays a pivotal role in HSP60-induced inflammation

**Fig. 7:**
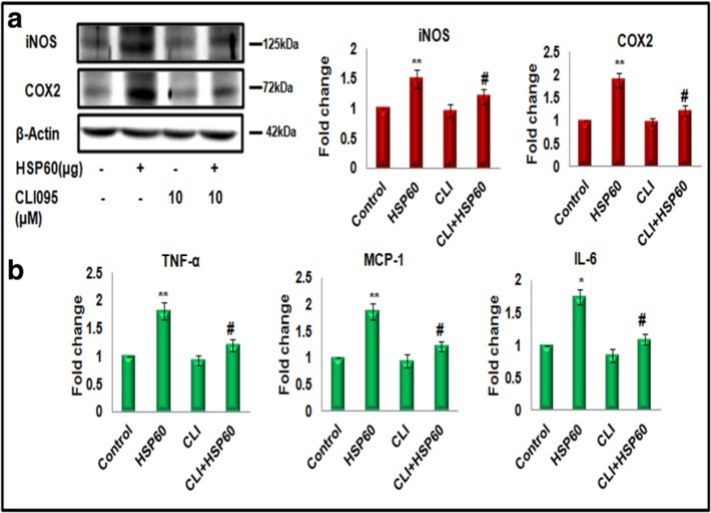
TLR4 plays a pivotal role in HSP60-induced inflammation in microglia. N9 cells were cultured in the presence or absence of 10 μM TLR4 inhibitor (CLI-095) 2 h prior to transfection of mouse HSP60 plasmid clone. **a** *Left panel* shows western blots illustrating effect of TLR4 inhibitor on iNOS and COX2 in the cells transfected with 4 μg mouse HSP60 plasmid clone or control pCMV6 plasmid, *right panel* represents the bar diagrams which reflect mean fold change in expression as compared to control. One hundred microgram protein was loaded for western blots of iNOS and COX2, and β-actin was used as a loading control. The blots are representative of three independent experiments. **b** CBA analysis of pro-inflammatory cytokines (TNF-α, MCP-1, and IL-6) also suggests a major involvement of TLR4 in HSP60-induced inflammation in microglia. Bar diagrams are mean fold change of three independent experiments with similar results. Data represented are mean ± SD of three independent experiments. *p < 0.05, **p < 0.01 in comparison to control values and ^#^ *p* < 0.01 in comparison to IL-1β treatment

As we found HSP60 to be secreted out in the extracellular milieu and interact with TLR4 to perform downstream signaling, we wanted to confirm whether inhibition of TLR4 signaling affects HSP60-induced inflammation in microglia. For this, we inhibited TLR4 signaling in microglial cells overexpressing HSP60 with specific TLR4 inhibitor (CLI-095, InvivoGen, 10 μM) and checked the levels of iNOS, COX2 (Fig. 7a), and pro-inflammatory cytokines (MCP-1, TNF-α, and IL-6) (Fig. 7b). As shown in Fig. 7a, b, levels of all these pro-inflammatory markers decreased significantly in presence of TLR4 inhibitor. Inhibition of TLR4 also reduces the levels of different pro-inflammatory molecules induced by HSP60 (Fig. 7a, b), thus further strengthening our hypothesis.

### Effect of HSP60 on mitogen-activated protein kinase (MAPK) phosphorylation

**Fig. 8:**
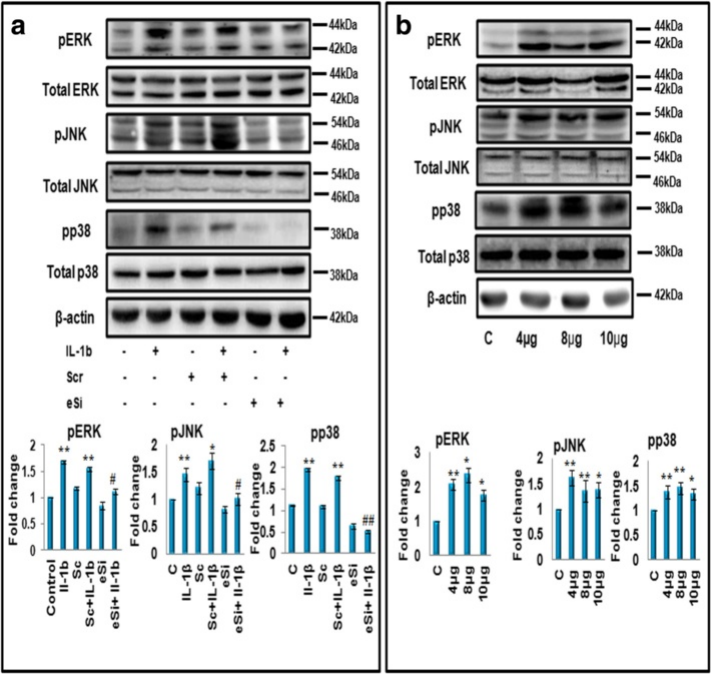
HSP60 regulates phosphorylation of MAPK effector molecules ERK1/2, JNK, and p38. **a** *Upper panel* depicts the western blot analysis of phospho- and total ERK1/2, JNK, and p38 in the N9 cells transfected with HSP60 esiRNA (at 6pM dose) or scrambled esiRNA (6pM). *Lower panel* shows the bar diagram which represents mean fold changes in the levels of phosphorylated forms of aforementioned proteins with respect to their total proteins. **b** Overexpression of HSP60 cDNA clone in microglial cells leads to increase in phosphorylation of all the three MAPKs at different doses of HSP60 cDNA clone. One hundred microgram protein was loaded for western blots, and β-actin was used as a loading control. Representative of three independent experiments is shown here. Graphs in lower panel represent mean fold change in the level of phosphorylation of MAPKs. The levels of phosphorylated proteins were normalized to their total proteins, respectively. Data represented are mean ± SD of three independent experiments. *p < 0.05, **p < 0.01 in comparison to control values and ^#^ *p* < 0.01 with respect to IL-1β-treated values

It has been well reported that IL-1β induces inflammation by activation of MAP kinase (MAPK) pathway (Additional file 1: Figure S3) in addition to phosphorylation of NF-κB [44, 45]. Additionally, according to some previous reports [46, 47], HSP60 acts as an antigenic protein and induces inflammation by inducing phosphorylation of MAPK proteins which lead to the execution of kinase pathway signaling mediated inflammatory response. Hence, we next investigated the effect of HSP60 expressed by microglia on the phosphorylation of MAPK proteins. For this, we knocked down HSP60 with specific eSiRNA and surprisingly, found significant decrease in the levels of phosphorylated forms of all three MAPK (ERK1/2, JNK, and p38) in HSP60 eSiRNA-treated cells as compared to cells transfected with non-specific scrambled eGFP eSiRNA (Fig. 8a). IL-1β treatment also only partially rescued the effect of HSP60 eSiRNA on phosphorylation of ERK and JNK but not in p38 MAPK (Fig. 8a). It seems that p38 is the specific target of HSP60. Further, we overexpressed HSP60 protein in N9 cells using mouse HSP60 cDNA clone at different doses, and immunoblot analysis revealed a significant increase in the phosphorylation of all three MAPK proteins in cells overexpressing HSP60 (Fig. 8b). The above results indicate that HSP60 regulates IL-1β-induced inflammation via activation of MAPK proteins.

### HSP60 induces inflammation in microglia via p38 MAPK activation

**Fig. 9:**
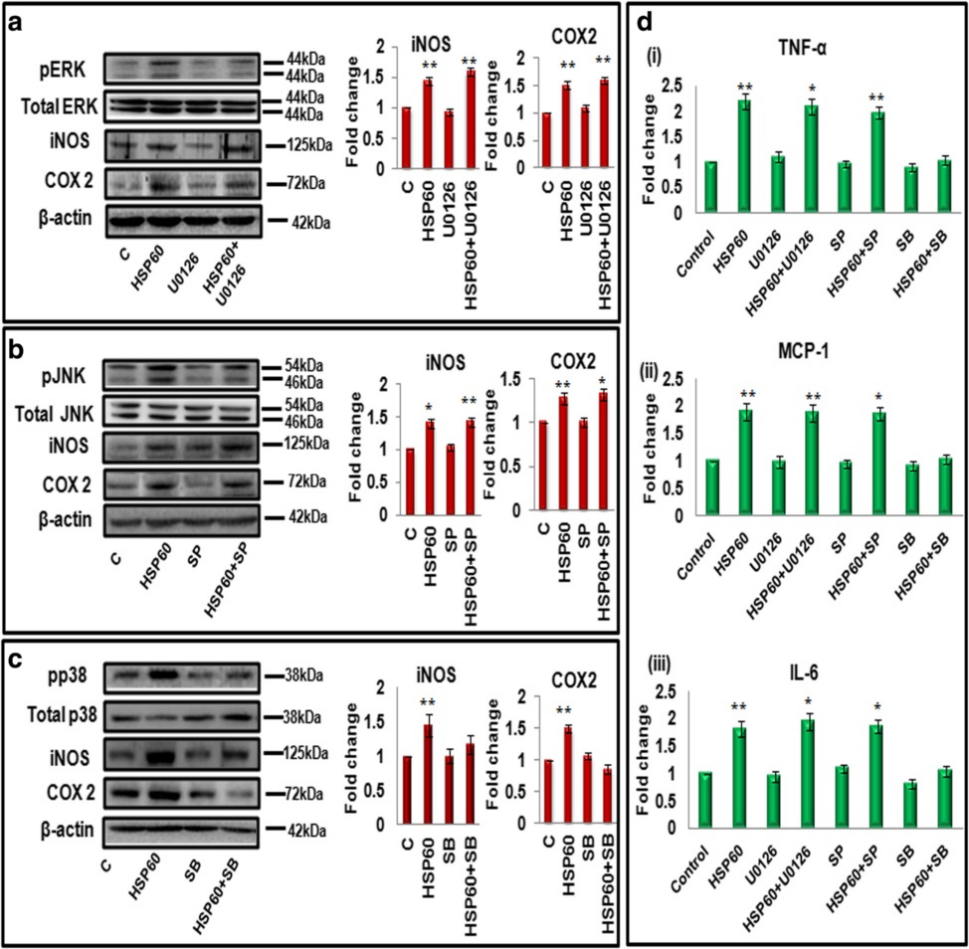
Effect of MAPK inhibitors on HSP60-induced inflammation in N9 microglial cells. N9 cells were cultured in the presence or absence of MAPK inhibitors; U0126 (10 μM), SP600125 (10 μM), and SB203580 (10 μM) 60 min prior to transfection of 4 μg of HSP60 cDNA clone and then incubated for 24 h. **a–c** The effect of ERK inhibitor U0126, JNK inhibitor SP600125 (SP), and p38 inhibitor SB0193 (SB) on phospho- and total ERK1/2 (**a**), on phospho- and total JNK (**b**), on phospho- and total p38 (**c**), respectively, and on pro-inflammatory molecules iNOS and COX2 (**a–c**). *Right panel* (**a–c**) shows bar graphs which represent mean fold change in iNOS and COX2 with respect to control, in different treatment conditions. The blots are representative of three independent experiments. One hundred microgram protein was loaded for western blots, and β-actin was used as a loading control. **d** Effect of specific inhibition of MAPKs on pro-inflammatory cytokines. CBA analysis of TNF-α, MCP-1, and IL-6 after treatment of N9 microglial cells with U0126, SP600125, and SB203580 inhibitors (*i*–*iii*). Data represented are mean ± SD of three independent experiments. *p < 0.05, **p < 0.01 in comparison to control values

To reveal the specific MAPK effector molecule which plays a crucial role in HSP60-modulated inflammation, we used specific inhibitors for these kinases. We treated N9 cells with specific MAPK inhibitors U0126 (10 μM), SP600125 (10 μM), and SB203580 (10 μM) for blocking phosphorylation of ERK pathway, JNK pathway, and p38 pathway, respectively, in addition to HSP60 cDNA clone and assessed the expression of pro-inflammatory enzymes (iNOS and COX2) and pro-inflammatory cytokines (TNF-α, MCP-1, and IL-6). To our surprise, blocking of ERK and JNK pathway in presence of HSP60 did not show marked decrease in the levels of iNOS, COX2, TNF-α, IL-6, and MCP-1 (Fig. 9a, b and d). These results suggest that ERK and JNK pathway do not show significant effect on HSP60-induced inflammation in microglial cells. In contrast, inhibition of p38 pathway showed marked decrease in inflammatory response of cells overexpressing HSP60 (Fig. 9c, d). This is reflected by the decrease of iNOS, COX2 and pro-inflammatory cytokines in the presence of p38 inhibitor which were induced by overexpression of HSP60 (TNF-α, MCP-1, and IL-6) (Fig. 9c, d). These results confirm that the downstream modulator which plays important role in HSP60-mediated inflammation is p38 MAP kinase which further aggravates the inflammatory process.

### MEK3/6: an important player in HSP60-induced inflammatory response in microglia

**Fig. 10:**
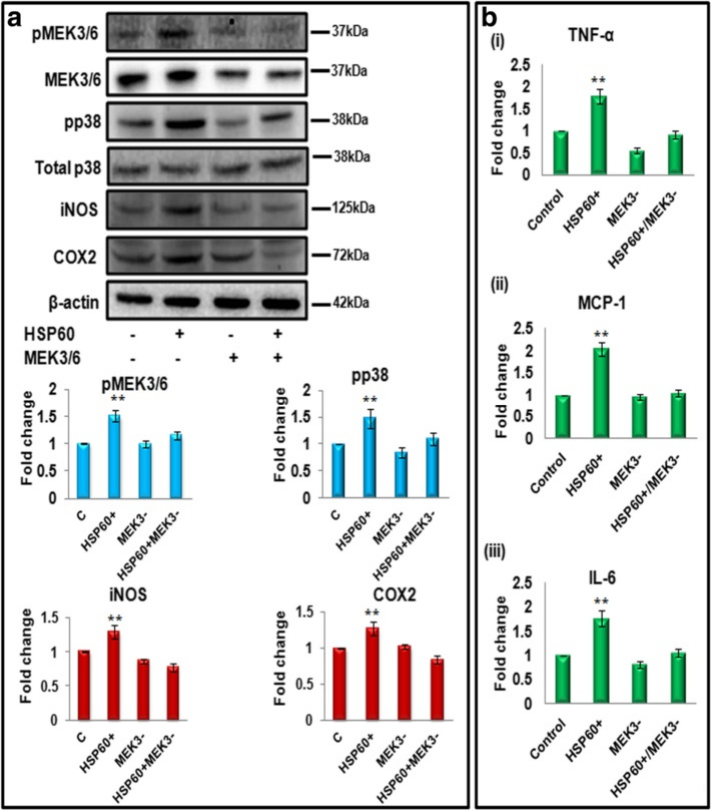
Role of MEK3/6 in HSP60-induced inflammation. N9 cells were transfected with 4 μg of HSP60 cDNA clone and/or, MEK3/6 specific eSiRNA (6pM) for 24 h, and the effect on inflammation was assessed by western blot (**a**) and cytokine bead array (**b**). **a** Western blot analysis of phospho-MEK3/6 and total MEK3/6, phospho- and total p38, iNOS and COX2 after inhibition of MEK3/6 and overexpression of HSP60. Blots are representative of three different experiments with similar results. One hundred microgram protein was loaded for western blots, and β-actin was used as a loading control. Graphs represent the mean fold change in the phosphorylation of ERK1/2, JNK, and p38 with respect to their respective total proteins and represents mean fold change in the expression of iNOS and COX2 with respect to control. **b** Effect of knockdown of MEK3/6 on pro-inflammatory cytokines. CBA analysis of TNF-α, MCP-1, and IL-6 after transfection of N9 microglial cells with HSP60 cDNA clone and/or, MEK3/6 eSiRNA (*i*–*iii*) Data represented are mean ± SD of three independent experiments. *p < 0.05; **p < 0.01 in comparison to control values

To further confirm the active involvement of p38 MAPK pathway in HSP60-mediated inflammation in microglia, we knocked down upstream molecule of p38 MAPK pathway, i.e., mitogen/extracellular signal-regulated kinase 3/6 (MEK3/6), which is responsible for causing phosphorylation of p38. Knockdown of MEK3/6 using specific siRNA specifically inhibits phosphorylation of p38 and overexpression of HSP60 only partially rescued the effect of MEK3/6 eSiRNA (Fig. 10a). Surprisingly, we observed a decrease in the pro-inflammatory enzymes (iNOS and COX2) as shown by western blotting (Fig. 10a) as well as pro-inflammatory cytokines (TNF-α, MCP-1, and IL-6) as shown by CBA (Fig. 10b). These results further streamline the signaling and confirm that HSP60 mediates inflammatory process in microglia by modulating MEK3/6 which phosphorylates p38 MAPK in a downstream pathway leading to inflammatory response.

## Discussion

Microglia, the resident immune cells of the central nervous system, receives signals from various stimuli ranging from pathogenic invasions, stress, toxins, and autoimmune diseases to neurodegeneration, and these signals act as the first warning that indicate disruption of normal cellular function in the organism and lead to the activation of microglia. Activated microglia further release endogenous inflammatory factors to activate other cells in nearby vicinity and the feedback cycle, thus proceeds to evoke acute or chronic inflammation. Microglial activation—which is marked by extensive proliferation, chemotaxis, and altered morphology—is the hallmark of neuroinflammation in several neurodegenerative diseases and pathological conditions of CNS [24]. Literature suggests that IL-1β, the master regulator of inflammation, induces microglial activation and plays a crucial role in the progression of chronic neurodegenerative diseases such as AD and PD as well as acute neuroinflammatory conditions including stroke, ischemia, and brain injury [18–20, 23]. However, the underlying molecular circuitry in IL-1β-induced microglial activation is still unexplored. In this study, we show that IL-1β causes activation of microglial cells by regulating the downstream signaling mediated via HSP60 to TLR4 to p38 MAPK. Our proteomics data revealed HSP60, the mitochondrial chaperone, as an important differentially regulated as well as highly interacted protein in IL-1β-stimulated N9 murine microglial cells, hence, we further stressed upon the role played by HSP60 in regulating IL-1β-induced inflammatory processes in microglia. We show that HSP60 secreted by microglia after IL-1β treatment also interacts with TLR4 receptor on microglia membrane. Using overexpression and knockdown experiments, we further reveal that HSP60 triggers microglia activation via TLR4-MEK3/6-p38 MAPK axis.

Several reports support that IL-1β secreted from activated microglia can activate other cells in the extracellular environment by activating different signaling pathways. Kim et al. reported that activated microglia secretes IL-1β which induces iNOS/NO in astrocytoma cells through p38 MAPK and NF-κB pathways [48]. Besides this, IL-1β induces the elevation of intracellular Ca^+2^ levels via the dual pathways of Ca^+2^ entry and Ca^+2^ mobilization [49]. Further, IL-1β has been reported to induce HSP60 expression in cultured human adult astrocytes [50]. This leads to the framework of our hypothesis that IL-1β-induced microglia inflammation may involve heat shock protein as an endogenous signal that can further relay inflammation via MAPKs inside the microglia.

**Fig. 11:**
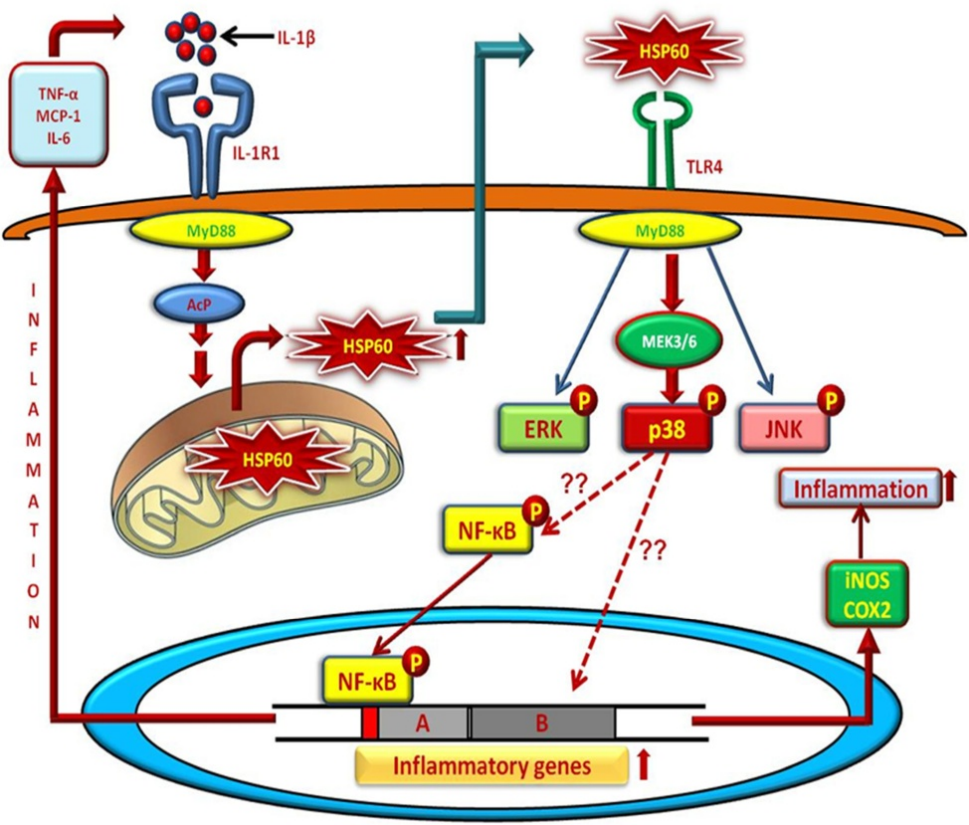
Schema of signaling pathway involved in IL-1β-induced inflammation in microglia. IL-1β induces inflammation by binding to its specific receptor IL-1R1 present on the cell surface and leads to the enhanced expression of HSP60 in microglia. HSP60 is secreted by the microglia into the surrounding and binds to TLR4 and further induces the inflammatory process by the activation of MEK3/6 which leads to increased phosphorylation of p38 MAP kinase pathway resulting in increased production of pro-inflammatory factors including TNF-α, MCP-1, and IL-6. HSP60, thus plays an important role in further increasing the intensity of inflammation by forming a feed-forward loop of inflammation in microglia

Based on our current findings, we hereby propose a model (feed-forward loop) of the signaling pathway leading to IL-1β-induced inflammation via HSP60 in microglial cells (Fig. 11). Stimulation of microglia by IL-1β induces binding of IL-1β ligand to its cognate receptor IL-1R1, and this increases the expression of HSP60 in the cytoplasm of cells. HSP60 is secreted out by the cells to give signals to possibly other cells in nearby vicinity to produce pro-inflammatory cytokines to combat the stressed situation; thus once induced, HSP60 regulates its own production in an autocrine and paracrine manner. This is in harmony with other reports where intracellular HSP60 has been shown to be secreted out of the cells [51]. Extracellular HSP60 then binds TLR4 receptor [31] which in turn is a part of the innate immune system and therefore secreted HSP60 expression positively correlates with the triggering of innate immune response by the production of pro-inflammatory molecules. Secreted HSP60 binds to TLR4 and upregulates the expression of TLR4 which further activates myeloid differentiation factor 88 (MyD88). MyD88 in turn leads to the phosphorylation of MEK3/6, a specific upstream modulator of p38 MAPK [52, 53]. Phosphorylation of MEK3/6 then specifically phosphorylates p38 MAPK which in turn increases the production of pro-inflammatory cytokines viz. TNF-α, MCP-1, and IL-6 and pro-inflammatory enzymes, i.e., COX2 and iNOS. In contrast to our study, Kilmartin et al. reported that treatment of monocytes with human HSP60 led to suppression of TNF-α production [54]. This difference can be attributed to different cell types and different cellular environments. A mitochondrial chaperone, i.e., HSP60 thus plays an important role in increasing the intensity of inflammation with its continuous production by forming a feed-forward loop of inflammation.

HSP60, in addition to an important molecular chaperone, has also been reported to have critical immunomodulatory roles. It has been found to be accumulated in the cytoplasm during apoptotic activation [55]. In contrast, HSP60 levels were reported to be significantly higher in cytoplasm of neuroepithelial tumors [56]. This chaperone has also been considered as a potential antitumor target [57]. Further, several evidences suggest the role of heat shock proteins in regulation of intracellular signaling [58–60]; however, the role of HSP60 in intracellular signaling leading to inflammation is sparsely explored. In the present study, HSP60 likely modulates intracellular signaling of IL-1β-induced inflammation. However, neuroinflammation is a complex process and, considering that several pathways are upregulated upon cytokine stimulation, therefore the role of other transcription factors and co-activators cannot be ruled out in IL-1β-induced inflammation.

IL-1β has previously been reported to orchestrate its function via its specific receptor, IL-1 receptor 1 (IL-1R1). However, our results clearly suggest that TLR4 is indeed playing a key role in IL-1β and HSP60-induced inflammation in microglia. Our results also propose that IL-1β may bind to TLR4, in addition to its cognate receptor IL-1R1, to exert its inflammatory effects in microglia, which is a novel finding and needs to be further explored. These findings are also in harmony with the two other recently published reports which claim that inhibition of TLR4 reduces vascular inflammation during hypertension [61, 62].

Literature suggests that p38 may act via several ways to induce the production of inflammatory cytokines. p38 may either act through MK2 to release TNF-α mRNA from translational arrest imposed by the ARE [63]. Another potential target of p38 is the redox-sensitive transcription factor NF-κB which is also one of the main transcription factors involved in TNF-α gene transcription. Since, we found increase in TNF-α and increased phosphorylation of p38 after HSP60 overexpression, hence, p38 MAPK might promote the release of inflammatory cytokines via a NF-κB dependent mechanism [64]. IL-1β has also been found to increase the expression of NF-κB in several studies [65]. However, p38 MAPK can also directly cause the production of pro-inflammatory cytokines (IL-1β, TNF-α, and IL-6) [66] and induction of enzymes such as COX2 [67] as well as p38 also modulates the expression of intracellular enzymes such as iNOS [68]. For defining these discrete functions and relationships of p38 to other molecules during IL-1β-induced inflammation, further investigation is warranted.

In this report, we firmly establish a molecular mechanism by which IL-1β leads to release of HSP60, which in turn activates microglia, the innate immune cells of CNS in a TLR4-MEK3/6-p38 MAPK-dependent manner. We thus speculate a model, in which neuroinflammation activates innate immunity through the release of HSP60 and activation of TLR4, leading to increased inflammatory response of microglia. Recently, intense research has been focused on immunomodulatory properties of heat shock proteins (HSP), including their role as adjuvant for vaccines in addition to their primary function [54]. Our results reveal a new potential mitochondria immunomodulatory chaperone, i.e., HSP60 that can be further evaluated as a therapeutic target for the management of inflammatory conditions of CNS as it induces inflammation by orchestration of inflammatory genes in response to IL-1β. Unlocking the signaling pathway underlying IL-1β-induced inflammation via HSP60-TLR4-p38 MAPK axis in microglia has for sure future implications for therapeutic management of neuroinflammatory disorders. Our study thus fills the gaps in current understanding of molecular circuitry of neuroinflammation and also provides a novel target as HSP60 for the treatment of various neuroinflammatory diseases. Future studies in this direction may provide conclusive answers.

## Conclusions

Observations from our present study suggest that IL-1β induces inflammation in microglia and alters the expression of various proteins, one of them is HSP60, which is a mitochondrial chaperone and plays a regulatory role in aggravating IL-1β-induced inflammation in microglia. IL-1β treatment not only increases the expression of HSP60 in microglia but it also leads to increased secretion of HSP60 from the microglia in the extracellular milieu. HSP60 then binds with TLR4 and induces inflammation in microglia by activating p38 MAPK via MEK3/6. In this study, we provide the first evidence of HSP60 as a new component of IL-1β-induced inflammatory network in microglial cells which further augments inflammation via TLR4-p38 MAPK axis.

## Additional file

Additional file 1:
**3 Supplementary figures and 2 Supplementary tables.** **Table S1.** List of proteins showing differential expression after IL-1β treatment in N9 microglia cells, identified by MS/MS analysis. **Table S2.** List of primers used for quantitative real time PCR (qRT-PCR) analysis. **Figure S1.** Pie chart showing the molecular functions of differentially expressed proteins in IL-1β treated N9 microglial cells. **Figure S2.** Protein-protein interaction network of the identified proteins. **Figure S3.** Effect of IL-1β on phosphorylation of MAPK effector proteins in vitro and *in vivo*. (DOCX 2526 kb)
