# Supplementary material for: HSP60 plays a regulatory role in IL-1β-induced microglial inflammation via TLR4-p38 MAPK axis
Source: J Neuroinflammation. 2016 Feb 2;13:27. doi: 10.1186/s12974-016-0486-x (PMC4736186; doi:10.1186/s12974-016-0486-x)
Supplement: Additional file 1: — 3 Supplementary figures and 2 Supplementary tables. Table S1. List of proteins showing differential expression after IL-1β treatment in N9 microglia cells, identified by MS/MS analysis. Table S2. List of primers used for quantitative real time PCR (qRT-PCR) analysis. Figure S1. Pie chart showing the molecular functions of differentially expressed proteins in IL-1β treated N9 microglial cells. Figure S2. Protein-protein interaction network of the identified proteins. Figure S3. Effect of IL-1β on phosphorylation of MAPK effector proteins in vitro and in vivo. (DOCX 2526 kb) [file 12974_2016_486_MOESM1_ESM.docx]

**Table-S1: Proteins showing differential expression after IL-1β treatment in N9 microglia cells, identified by MS/MS analysis of gel excised spots**

| **Spot**  **No.** | **Protein ID/ Accession no.** | **Matched peptides** | **Ion**  **Score** | **%**  **Cov.** | **M score** | **MW**  **(theor./**  **observed)** | **pI** |
| --- | --- | --- | --- | --- | --- | --- | --- |
| N1 | **Aldehyde Dehydrogenase**  **(EDL19720.1)** | K.VAFTGSTEVGHLIQVAAGSSNLK.RR.TFVQENVYDEFVE  R.SR.VVGNPFDSR.T | 142  131  43 | 36 | 697 | 56502/  26400 | 7.53 |
| N2 | **Heterogeneous nuclear ribonucleoprotein H**  **(NP_067485.1)** | R.EGRPSGEAFVELESEDEVK.LR.YVEVFK.SK.HTGPNSPDTANDGFVR.L | 100  21  115 | 59 | 846 | 49168/  51900 | 5.89 |
| N3 | **HSP 60**  **(NP_034607.3)** | R.TVIIEQSWGSPK.VK.LVQDVANNTNEEAGDGTTTATVLAR.SK.GANPVEIR.R | 39  152  37 | 36 | 945 | 60917/  56000 | 5.91 |
| N4 | **ATP synthase subunit β**  **(NP_058054.2)** | R.LVLEVAQHLGESTVR.TR.TIAMDGTEGLVR.GK.VLDSGAPIKIPVGPETLGR.I | 106  9  80 | 26 | 445 | 56265/  26000 | 5.19 |
| N5 | **Stress-70 Protein**  **(NP_034611.2)** | R.TTPSVVAFTADGER.LR.RYDDPEVQK.DK.NAVITVPAYFNDSQR.Q | 44  12  117 | 27 | 554 | 73416/  70000 | 5.81 |
| N6 | **Actin, cytoplasmic-2**  **(NP_033739.1)** | K.AGFAGDDAPR.AK.IWHHTFYNELR.VR.VAPEEHPVLLTEAPLNPK.A | 80  77  103 | 40 | 656 | 41766/  40300 | 5.31 |
| N7 | **Heme binding protein 1**  **(EDL10544.1)** | K.FATVEVTDKPVDEALR.EK.EADYVAHATQLR.TR.RNEVWLVK.A | 32  89  7 | 24 | 145 | 21053/  23000 | 5.18 |
| N8 | **Tropomyosin α-3**  **(NP_071709.2)** | K.AADAEAEVASLNR.RR.RIQLVEEELDR.AR.IQLVEEELDR.A | 70  23  73 | 32 | 503 | 32974/  30300 | 4.68 |
| N9 | **Nucleophosmin**  **(ADA57701.1)** | K.VDNDENEHQLSLR.T | 76 | 16 | 90 | 32540/  28500 | 4.62 |
| N10 | **Nucleophosmin**  **(ADA57701.1)** | K.VDNDENEHQLSLR.T | 89 | 18 | 108 | 32540/  27500 | 4.62 |
| N11 | **Vimentin**  **(NP_035831.2)** | R.SLYSSSPGGAYVTR.SK.VELQELNDR.FK.ILLAELEQLK.G | 125  50  59 | 60 | 903 | 53655/  200000 | 5.06 |
| N12 | **HSP-75**  **(NP_080784.1)** | K.DISEFQHEEFYR.YR.YIAQAYDKPR.FR.GVVDSEDIPLNLSR.E | 113  67  56 | 20 | 547 | 80159/  72000 | 6.25 |
| N13 | **NADH ubiquinone oxidoreductase**  **(EDL00182.1)** | R.FCYHER.LR.FASEIAGVDDLGTTGR.GR.FEAPLFNAR.I | 13  93  45 | 24 | 281 | 79726/  76000 | 5.51 |
| N14 | **Proteasome subunit alpha type 1**  **( NP_002777.1)** | R.NQYDNDVTVWSPQGR.IK.ILHVDNHIGISIAGLTADAR.LR.FVFDRPLPVSR.L | 103  72  12 | 24 | 204 | 29528/  29400 | 6.00 |
| N15 | **T-complex protein 1 unit epsilon**  **(NP_031663.1)** | R.IADGYEQAAR.IK.IAILTCPFEPPKPK.TR.WVGGPEIELIAIATGGR.I | 20  24  137 | 22 | 258 | 59586/  62000 | 5.72 |
| N16 | **NADH ubiquinone oxidoreductase**  **(EDL00182.1)** | R.FCYHER.LR.FASEIAGVDDLGTTGR.GK.VDSDNLCTEEIFPTEGAGTDLR.S | 23  93  112 | 36 | 466 | 79726/  76000 | 5.51 |
| N17 | **ATP synthase subunit β**  **(NP_058054.2)** | R.LVLEVAQHLGESTVR.TK.IPVGPETLGR.IR.IMNVIGEPIDER.G | 101  71  45 | 33 | 543 | 56265/49000 | 5.19 |
| N18 | **Vimentin**  **(NP_035831.2)** | R.SYVTTSTR.TR.TYSLGSALRPSTSR.SR.SLYSSSPGGAYVTR.S | 13  3  91 | 74 | 1730 | 53655/57000 | 5.06 |
| N19 | **HNRNP c1/c2**  **(NP_001164455.1)** | R.VFIGNLNTLVVK.KK.GFAFVQYVNER.NR.MIAGQVLDINLAAEPK.V | 63  87  91 | 37 | 498 | 34364/41000 | 4.92 |
| N20 | **Gelsolin**  **(AAH23143.2)** | K.EPGLQIWR.VK.HVVPNEVVVQR.LR.SEDCFILDHGR.D | 31  63  22 | 12 | 146 | 85888/  84200 | 5.83 |
| N21 | **Glutaredoxin-3**  **(AAH87885.1)** | R.HVSSGAFPPSTNEHLK.EK.HNIQFSSFDIFSDEEVR.Q | 42  102 | 14 | 161 | 37754/  40800 | 5.42 |

**Table S2: List of primers used for quantitative real time PCR (qRT-PCR) analysis**

| **HSP60 (Forward primer)** | **5′ GCAGAGTTCCTCAGAAGTTGG -3′** |
| --- | --- |
| **HSP60 (Reverse primer)** | **5′- GCATCCAGTAAGGCAGTTCTC -3′** |

**
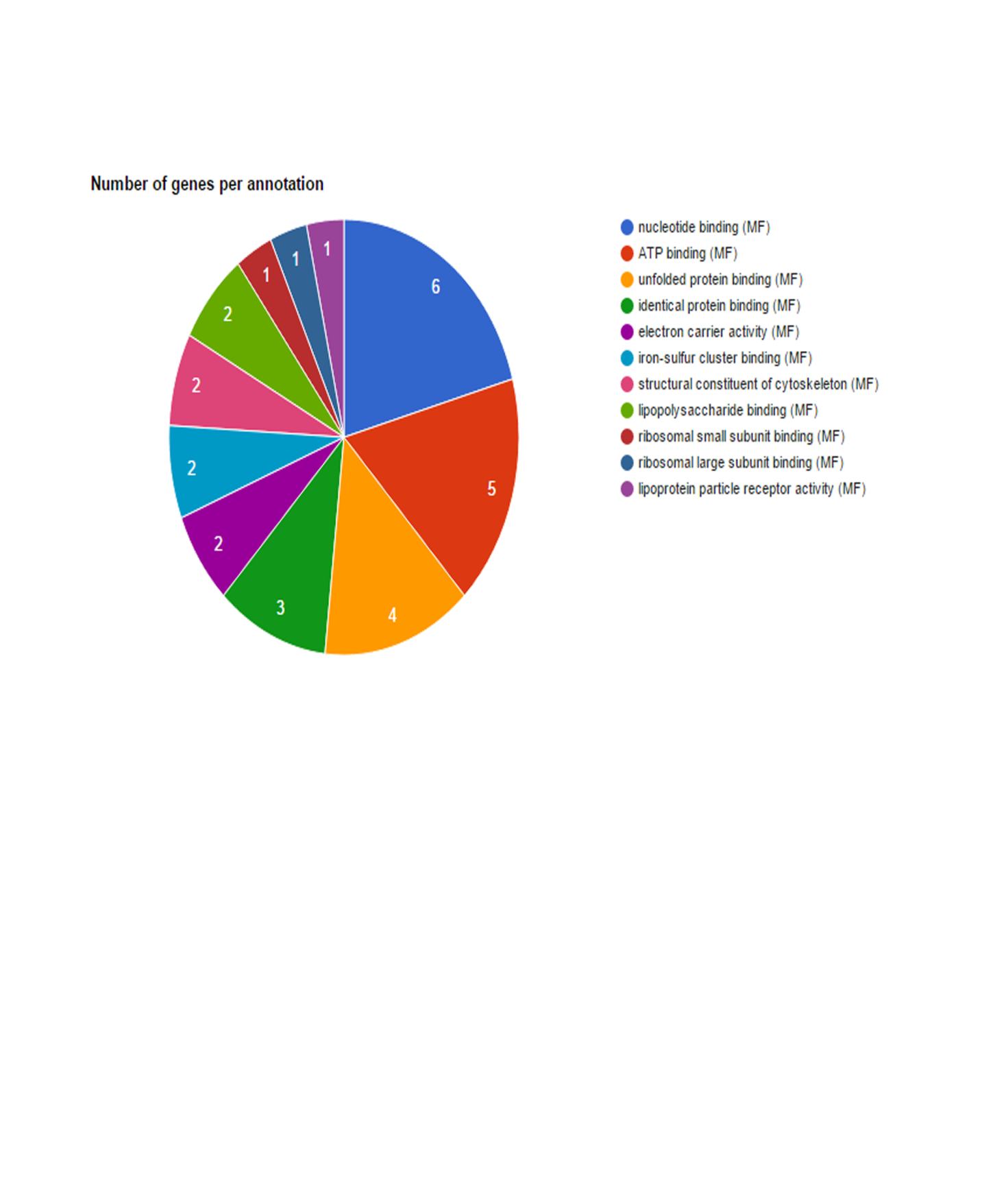
**

**Figure S1:** Pie chart showing the distribution of differentially expressed proteins in IL-1β treated microglia N9 cells into different molecular functions. The list of differentially expressed proteins was uploaded into the GeneCodis3 software, and the list of significantly enriched molecular functions was generated. The number in each quadrant represents number of proteins in each molecular function.


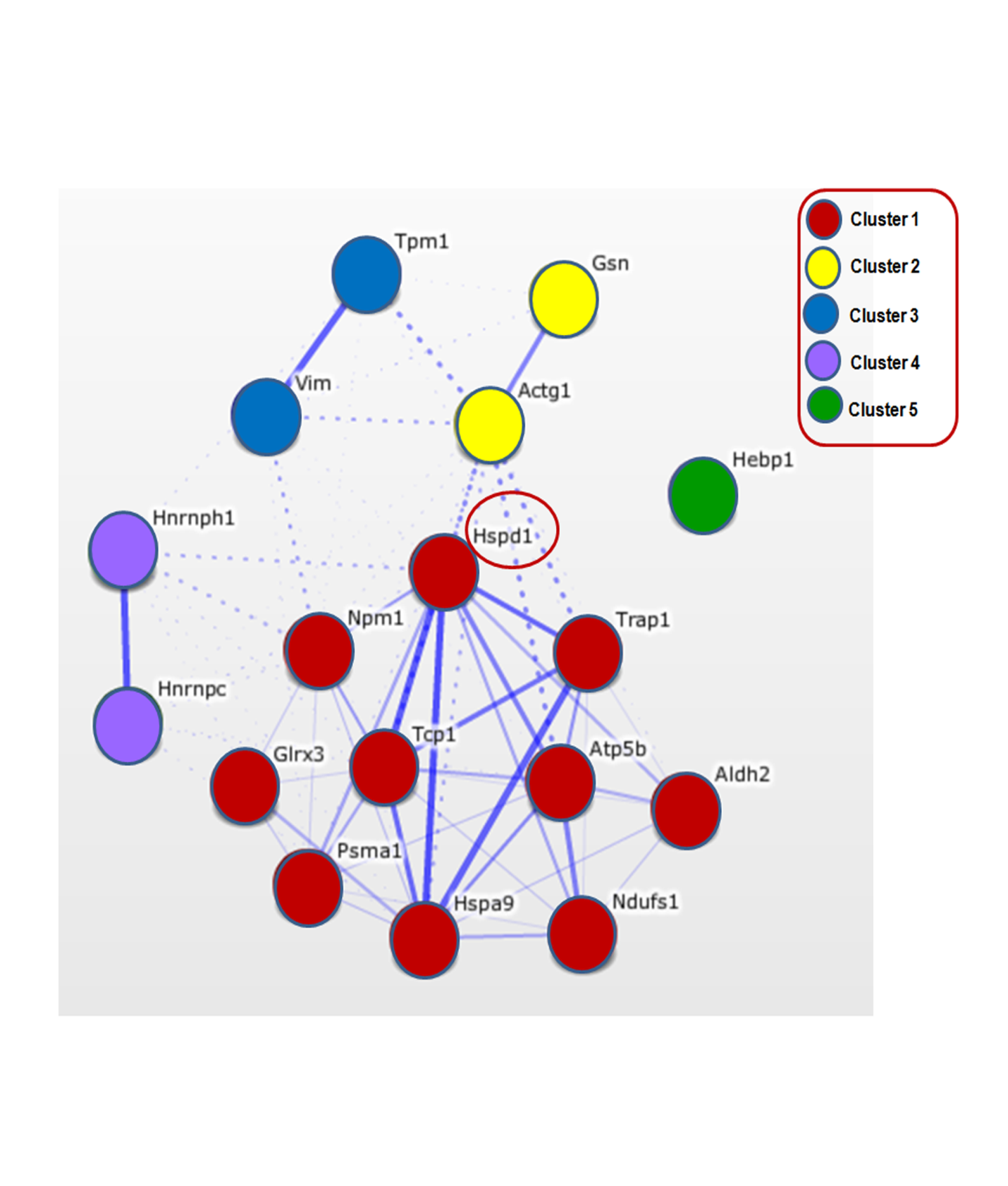


**Figure S2: Protein-protein interaction network of the identified proteins.** The interactome was generated from the identified with the help of STRING database, using low confidence (0.150) parameter, in order to identify highest possible connections. Further, highest degree of MCL clustering was applied to identify different clusters, out of which HSP60 (Hspd1, encircled) is present in the biggest cluster and has highest number of connections.


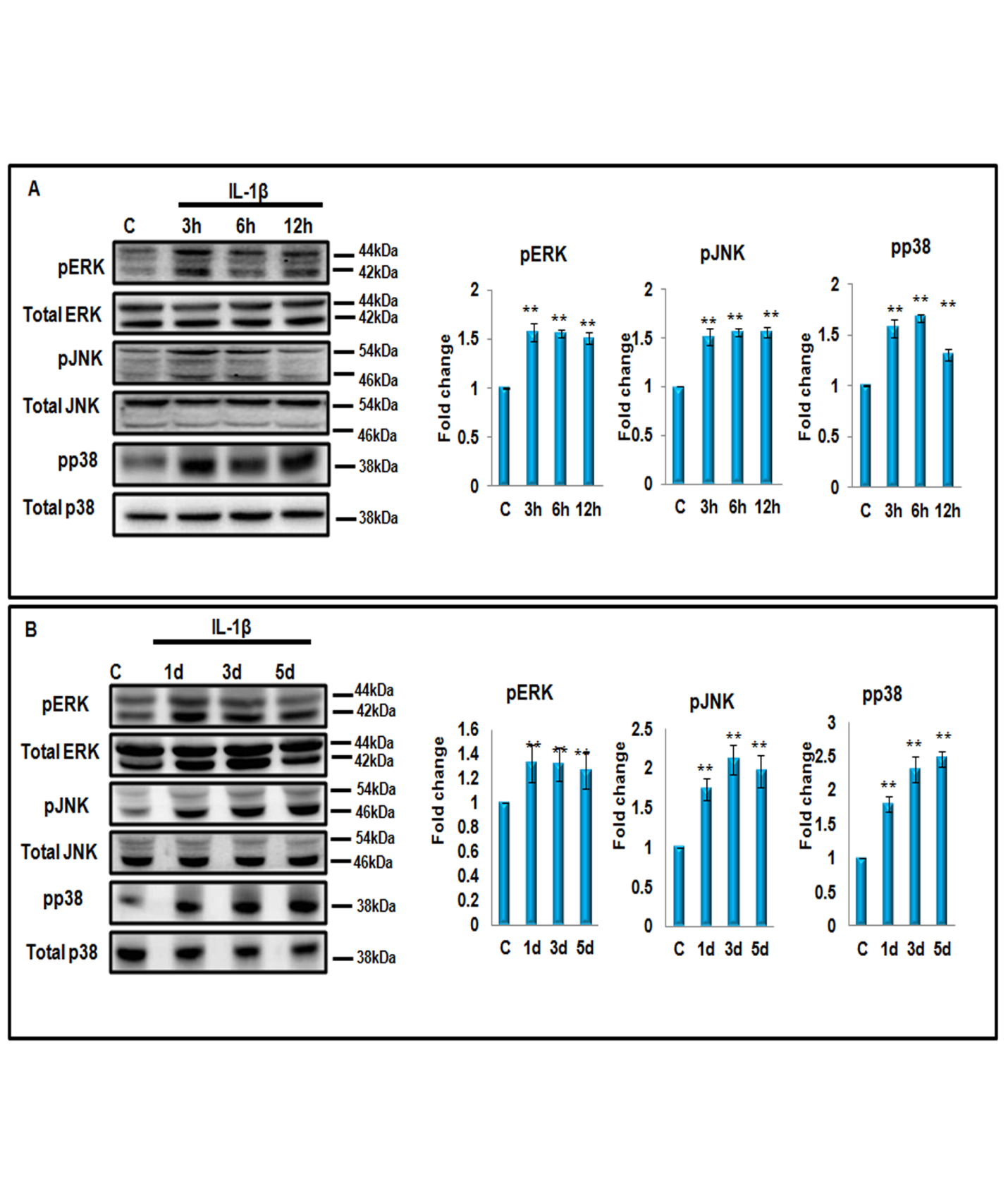


**Figure S3: Effect of IL-1β on phosphorylation of MAPK effector proteins** IL-1β induces phosphorylation of all three types of MAPK (ERK, JNK and p38) in a time dependent manner both in vitro and in vivo. **A.** Left panel depicts the western blot analysis of phospho- and total ERK1/2, JNK and p38 in the cells treated with 5 ng/ml IL-1β for different time points (3, 6 and 12 hours) in N9 murine microglia cells. Right panel shows the bar diagram which represents fold changes in the levels of phosphorylation in ERK1/2, JNK and p38. The levels of phosphorylated proteins were normalized to their total proteins respectively **B.** Left panel depicts the western blot analysis of phospho- and total ERK1/2, JNK and p38 in the cells treated with 10 ng/g body weight of IL-1β dissolved in 1X phosphate-buffered saline (PBS) every 24 h for different durations (1, 3 and 5 hours) in BALB/c mice. Right panel shows the bar diagram which represents fold changes in the levels of phosphorylation in ERK1/2, JNK and p38. The levels of phosphorylated proteins were normalized to their total proteins respectively. Data represented are mean ± SD of three independent experiments. *p < 0.05, ** p < 0.01 in comparison to control values.
